# Developing an Ethical Evaluation Framework for Coercive Antimicrobial Stewardship Policies

**DOI:** 10.1093/phe/phae005

**Published:** 2024-04-23

**Authors:** Tess Johnson

**Affiliations:** Ethox Centre, University of Oxford, UK

## Abstract

Antimicrobial resistance (AMR) has been declared one of the top ten global public health threats facing humanity. To address AMR, coercive antimicrobial stewardship policies are being enacted in some settings. These policies, like all in public health, require ethical justification. Here, I introduce a framework for ethically evaluating coercive antimicrobial stewardship policies on the basis of ethical justifications (and their limitations). I consider arguments from effectiveness; duty of easy rescue; tragedy of the commons; responsibility-tracking; the harm principle; paternalism; justice and development; a precautionary approach; and professional duties. I consider how these justifications might form the basis for developing a comprehensive ethical framework, and the need for this to be context-specific and aligned with the priorities, evidence and needs of the particular jurisdictions in which a policy is to be enacted. I demonstrate how the ethical justifications might be used by reference to an example policy of the EU ban on the use of certain human-critical antibiotics for livestock, before concluding with challenges for further development of the framework.

## Introduction

Antimicrobial resistance (AMR) is described by the World Health Organization as ‘a global health and development threat’ that ‘requires urgent multisectoral action in order to achieve the Sustainable Development Goals (SDGs)’ (World Health Organization, 2021). AMR could cause 10 million deaths per year by 2050 if no action is taken (O’Neill, 2016), with financial losses amounting to USD 6.1 trillion per year (World Bank, 2017). Although it has been declared one of the top ten global public health threats facing humanity, however, AMR has also been called a silent pandemic and a hidden threat, with low public awareness of the problem.

AMR is exacerbated by excessive use of antimicrobials in some industries, health systems, or areas of the globe, and by a lack of access to effective antimicrobials in others. AMR is also, to some extent, inevitable: whilst it is exacerbated by use of antibiotics, even without human use microbes would adapt to natural antimicrobial agents in their environments and gradually become resistant. Because of these factors, we cannot treat AMR as a single, blanket problem with a lone solution. Rather, there is room for fruitful discussion concerning the multiple approaches that might be taken to address the problem. Typically, actions to address AMR are divided into those that concern innovation to develop new antibiotics or alternative therapeutics or preventives, and antimicrobial stewardship (AMS) measures, which aim towards appropriate use of existing antibiotic stocks—sometimes through coercive policies.

From a policymaker’s perspective, where voluntary AMS measures fail or are less effective than coercive alternatives, coercive policies such as bans or taxes on antibiotic use in some sectors, capping doctors’ prescriptions, or eliminating over-the-counter antibiotic supply chains might be attractive—for instance, they may seem likely to be more effective or fair. Both effectiveness (at securing benefit) and fairness could be ethical considerations in favour of a coercive policy. Yet, there are often clear points against a coercive policy, as well. For instance, in jurisdictions with ethical traditions that value peaceful relations and consensus, coercive policies may undermine these values (Ujomudike, 2016); peaceful relations might be compromised through the enforcement of penalties for non-compliance, and coercive policies may be less likely than voluntary measures to be the result of community consensus (though not in all cases). Similarly, in the liberal democratic tradition, coercive action might seem ethically problematic where it undermines other values, such as by restricting freedom.

Because there are clear ethical points both for and against prospective coercive action, and policymakers must justify any action taken, it is important that they consider the full range of ethical justifications behind coercive AMS policies. Not all ethical justifications for a coercive action will be particularly strong, or appropriately used to justify a particular AMS policy. That will depend on the context-specific limitations regarding when the premises of the ethical justifications are supported. Still, assessing the full range of justifications and their strength and applicability to prospective coercive AMS policies is important both to policymakers and the public, for a number of reasons. First, articulating strong justifications for AMS policies clarifies the trade-offs involved between different values in the course of taking a particular policy action. Second, it ensures transparency and accountability for policy choices (Schaefer *et al*., 2023). What’s more, policymakers with multiple ethical justifications for a policy to hand can also appeal to multiple perspectives and stakeholders. That is, where there are multiple *pro tanto* or alternative ethical arguments available for the action they have taken that do not conflict with each other (McInnes and Mahler, 2014), different groups within the public, with different sets of values, may be appealed to accept coercive policy at the same time. Related to this reason for considering ethical justifications, the public should also be in possession of a list of ethical justifications and the limitations of each, to better evaluate policymakers’ reasoning for chosen policies. Public justification is secured where the public accept the reasoned arguments given for an action, and that can only be achieved where both parties (policymakers and the public) have access to arguments or justifications to evaluate them.^1^ Finally, to prevent instrumentalisation of ethical justifications for political purposes, it is important that the public should have access to the means to ethically evaluate policy choices, and it should be emphasised that the core purpose of the framework I introduce here is to facilitate better ethical evaluation of potential justifications for prospective coercive AMS policies.

In what follows, I present the beginnings of an ethical evaluation framework for coercive AMS policies (hereafter, ‘the framework’). No work that I am aware of has yet brought together ethical arguments for coercive AMS policies from the existing ethics literature, considered their limitations and discussed developing them into a framework for analysing policy. This is despite ongoing calls for more and better ethical reflection in policymaking (Degeling *et al.* 2021; Yen and Cutrell 2021). This paper fills the gap. It is not a fully developed framework, for reasons I will outline later. Rather, it is an indication of how one could be developed, using already-available ethical justifications. In the following sections, I first offer some background on coercion, and then evaluate each of the arguments that might be used to justify coercive policy to address AMR. A core aspect of this framework is the systematic evaluation of the concepts listed, such that it is clear how the concepts are limited by the conditions for accepting their premises, and that they cannot ethically justify any coercive AMS policy in any context. I go on to indicate how a developed form of the framework could be applied in policy decision-making, using the example of recent EU policy on antibiotic use in livestock. Finally, I consider challenges for further development of the framework.

## Background on Coercion

Identifying what is included in the concept of coercion will help to set the stage for justifying coercive policies. In doing so, I will contrast two traditional accounts of coercion, which emphasise different aspects, respectively: threat of negative consequences in Robert Nozick’s pressure account of coercion, and reduction of freedom of choice in an authority account. I employ the latter account going forward, as it encompasses actions that we typically believe to be coercive that are excluded by the narrower pressure account.

One of the more influential definitions of coercion was introduced by Nozick (1969). He holds that a coercer coerces a coercee where the coercer successfully employs a threat to keep the coercee from performing an action. For instance, coercion occurs where a fine is threatened for drink driving. Threat is used to pressure another’s will to comply with the coercer’s.

A second account of coercion doesn’t allow for coercive action only through the use of verbal threat, but also through physical force or the elimination of the coercee’s alternative options by the coercer. Hobbes (1929 [1651]), Locke (2015 [1689]), and Kant (1996 [1797]) have all discussed coercion from the perspective of state authority to restrict some individuals’ freedoms (for instance, the freedom to smoke in a restaurant) in order to protect others’ freedoms (for instance, the freedom to breathe unpolluted air). According to Anderson (2010), this kind of coercion is notable for its exploitation of power differentials present in the relationships between states and subjects, or parents and children, where one is an authority figure. This account allows for coercion to occur either via the threat of a negative consequence by the authority, or by the implementation of that negative consequence, thus encompassing, for instance, the enforcement of a fine for drink driving, not only the threat of it.

Using this latter authority account of coercion, Joel Feinberg (1984) holds that whenever a legislator faces a choice between infringing a citizen’s liberty or leaving it intact, they should maintain liberty. This is due to a presumption common in liberal democratic societies, that freedom is to be valued, and, thus, that those who would restrict it bear a burden of justification (Feinberg, 1984). This may make it seem tempting to employ a normative account of coercion, where coercion is a *pro tanto* wrong. However, liberty is not the only value we ought to promote, and as I have noted, policies that employ coercion may be more effective or fair than non-coercive alternatives. I hold, accordingly, that a normatively neutral, authority account of coercion is the most appropriate in the context of public health policymaking.^2^

In the context of public health policy to address AMR, coercive measures for justification might include caps on doctors’ antibiotic prescriptions, prohibitions on farmers giving antimicrobials important for human use to their livestock and licensing and regulation of the import of antibacterial household cleaning products, all of which would be included in authority accounts of coercion. Sometimes, a non-coercive AMS policy may lead to individuals or communities coercing each other. I am only concerned here with directly coercive AMS policies, but note that such policies—for example, educational material promoted in kindergartens that leads parents to pressure each other not to give their children antibiotics—also require ethical scrutiny.

I turn next to mapping ethical concepts relevant to coercive AMS policy as they have been presented in the most prominent bioethics literature to date, which might form the basis of a framework for ethically evaluating prospective coercive AMS policy. The literature was not systematically reviewed in the development of the list of concepts below. Instead, a snowballing approach was taken for an informal literature review, and the concepts are further developed and delimited through my own analysis. It is this evaluative component that forms the basis of a novel contribution to the literature on AMR: evaluation of these ethical concepts as justifications for coercive measures reveals strengths, weaknesses and requirements for evidence that have not yet been explored and that highlight discrepancies between current vs appropriate usage of the concepts as ethical justifications by policymakers.

## Possible Justifications in an Ethical Evaluation Framework for Coercive AMS Policies and Their Limitations

### Effectiveness

The first justification a policymaker might consider for a proposed coercive AMS policy might be effectiveness. For instance, a policy that would require hospitals to undertake targeted patient screening for Methicillin-resistant *Staphylococcus aureus* (MRSA) might be justified using an argument from effectiveness.^3^ The overall claim would be that targeted hospital screening for MRSA, say, is the most effective feasible policy measure to address AMR on a population level. This argument relies on several premises. First, the premise that AMR poses significant, credible threat of harm to human and animal health and environmental resilience. Second, in some forms of the argument there is a comparative premise that might say that, all else equal, this threat is better mitigated by this coercive policy than by an alternative. To accept the premises of this argument we would require evidence of the harms of MRSA in hospitals, and evidence that conducting hospital screening upon patient admission is effective at producing information that can prevent the spread of hospital-acquired MRSA infections (through the subsequent infection prevention and control measures), and more so than alternative (less coercive) measures. If the premises of the argument are met, then the effectiveness of a coercive action may be a compelling argument in its favour.

The argument is limited, however, by a few conditions. First, it may be difficult for the empirical premises to be met. Gathering evidence on both the seriousness of the threat, and causal pathways between particular activities or events and worsening/ or mitigating resistance can be difficult (Johnson and Matlock, 2023). What’s more, even if these conditions are met, pursuing a more effective policy might come at a different cost. For instance, it may unfairly restrict the activities of particular people who already experience the brunt of structural inequalities in a society (Pokharel *et al.*, 2024). Finally, a policy that looks more effective on paper may, in fact, be less effective than alternatives if it has insufficient penalties or enforcement to ensure compliance, or no mechanism for verifying compliance (Gostin *et al*., 2023).

Overall, effectiveness can be a strong justification for coercive AMS policy in contexts where there is good evidence that the premises are fulfilled, particularly where there are high levels of MRSA transmission in hospitals, and adequate resources to support screening and changes in infection prevention measures. However, this justification may still be outweighed by other costs associated with the policy (including compromising other values).

### Duty of Easy Rescue

Another justification a policymaker might consider for a coercive AMS policy is the duty of easy rescue. Coercive policies are often held to be more justifiable regarding their impacts on individuals if they merely enforce individuals’ existing, independent moral obligations. One possible moral obligation is a ‘duty of easy rescue’, first developed by Peter Singer in the context of duties to donate to charity (Singer, 1972). The concept has since been adapted to the population level, and to the AMR context, where it is employed as an argument to justify coercive actions including restricting or discouraging patients’ use of antibiotics for mild and self-limiting infections (Giubilini and Savulescu, 2020) and as an argument to justify prohibiting certain uses of human-critical antibiotics in livestock (Johnson, forthcoming). The core claim is that an individual or group has an obligation to help another where they can do so at a cost to themselves that is reasonably bearable. For instance, an individual may have an obligation not to use antibiotics for a mild and self-limiting infection, in order to contribute to protecting others from suffering the harms of AMR. For this to constitute a strong ethical justification, the premises would need to hold that: patients forgoing antibiotics do in fact benefit others by preserving antimicrobial effectiveness or reducing the likelihood of their developing and spreading a resistant infection; and that it is reasonably bearable for them (and as such, a case of ‘easy rescue’) to do so.

Again, this argument is limited in that it relies on evidence backing up these two premises, and it may be that other factors render the costs associated with having a mild and self-limiting infection not reasonably bearable for individuals. Perhaps, for instance, some of these individuals have care responsibilities for others that they could not perform if even mildly ill, meaning that forgoing antibiotics and suffering a mild and self-limiting infection is more burdensome for them than it first appears. Another limitation is a counterargument to easy rescue. ‘Rescue’ implies urgency and threat of extreme or disastrous harm. Some may question whether AMR fulfils the conditions to be considered a rescue scenario, given its chronic nature and the lack of certain harm to any particular individual (Krockow and Tarrant, 2019). Finally, this justification might be considered ambiguous: on one interpretation, it may demand much of agents if the cost of action is considered in comparison to the benefit to others. On another interpretation, it may demand too little of agents if we set a low bar for what is ‘reasonably bearable’ as a cost and do not compare it to the benefit others receive from the action, but rather, consider it in absolute terms.

The fact that the duty from easy rescue relies on an independent moral obligation individuals already have makes it quite compelling as a justification for coercive stewardship, as long as other conditions are satisfied. The satisfaction of those conditions is more likely in contexts where AMR poses a greater and more predictable current threat to members of the population, and where individual behaviours (rather than systems and structures) contribute to and may reduce the risk of harm to others.

### Tragedy of the Commons

A third argument for coercive action might be the ‘tragedy of the commons’. Alberto Giubilini (2019) treats antimicrobial effectiveness as a globally shared resource, a ‘commons’ to which we all contribute by not overusing antibiotics and ensuring they are accessible when truly needed. We might have a collective, group-level obligation and subsidiary individual-level obligations to contribute to protecting such commons, justifying coercion where we do not or cannot coordinate to protect the commons. Giubilini presents a scenario where the commons is overused and thus degraded, based on Garrett Hardin’s (1968) original problem. It is difficult to establish individual accountability in commons scenarios, and as such, group-level coercive action may be required, offering a justification for, in Giubilini’s example, taxing antibiotic use in high-income countries (HICs) in cases where infections are mild and self-limiting. The commons argument might also be used to justify measures such as prohibitions on patients taking antibiotics for mild and self-limiting conditions. The goal of taxation is both to reduce use, and to create a source of income for expanding the commons, by funding research and development of new antibiotics. Either way, it is in the public interest to implement taxation and ensure the commons is protected. In such cases, individuals either forgoing antibiotics or paying taxes on their use of antibiotics contribute to the commons. The argument relies on a premise that antimicrobial effectiveness constitutes a common pool resource, meaning that, among other things, it must truly be globally shared (which seems at least plausible, considering how resistance develops across multiple pathogens, and how treatments often rely on the same limited list of globally available antibiotics). It also relies on a premise surrounding the effectiveness of taxation at reducing use or increasing funding for research and development of new antibiotics.

These requirements show the significant limitations of this justification. Consider that taxation will not expand the commons unless funds are used to support antimicrobial drug innovation, which is not guaranteed. Consider, also, that coercive measures justified by the argument (including taxation) may themselves have inequitable effects regarding who is likely to be discouraged from taking antibiotics (those financially worse off) and who is not (those financially better off). Or consider that if the commons are not protected initially, then they may cease to be globally shared—that is, antimicrobial effectiveness may only be preserved in particular regions or sectors. In that case, the tragedy of the commons argument may no longer be valid. Finally, consider another way that antimicrobial effectiveness may not technically fulfil the requirements to be considered a common pool resource, in that particular individual uses of antibiotics may not necessarily contribute to degrading the commons. Rather, each use is only probabilistically harmful (depending on whether an individual ends up hosting and spreading a resistant pathogen) (Jamrozik and Heriot, 2022).

This argument in favour of coercive action is relatively limited due to the difficulties fulfilling its premises. In particular, it is important for this argument that there is global coordination of AMS actions. Where this requirement is fulfilled alongside the others, however, it may be appropriate for justifying coercive state AMS policy.

### Responsibility-Tracking

Policymakers might also consider penalties for past bad actions to be appropriately part of coercive AMS policy. For accountability-type responsibility, we might think it appropriate to punish those who have done something wrong, which might also serve to change future behaviour. For task-type responsibility, we might think it appropriate to require those who have the power to avert a future expected bad outcome to take on the task. In the case of AMR, there are some groups or individuals who might be held particularly accountable for bad outcomes—perhaps, farmers in HICs who continue to use antibiotics important for treating infections in humans to increase their livestock growth. There are also groups who we might believe have greater capacity than most to avert future bad outcomes—perhaps, pharmaceutical companies involved in drug development. In these cases, we might think that any coercive action that occurs is better justified insofar as it ‘tracks’ one or both of these kinds of responsibility by imposing burdens or restrictions on those who are accountable and imposing action requirements on those who have capacity to make change (Johnson and Matlock, 2023).

This argument, too, may run into problems in certain contexts. Moral (as opposed to merely causal) responsibility requires a number of conditions to be met, including that agents act with shared intent, that they are aware of the potential outcomes of their actions, and that they have feasible alternatives available (Shoemaker, 2015). For instance, whilst farmers using important antibiotics for growth promotion in HICs may be morally responsible, it’s unclear that farmers in low- and middle-income countries (LMICs) always have feasible alternatives. Although important antibiotics are used in farming more frequently in some LMICs, this may be required in some settings to prevent disease in livestock living in crowded conditions. In other settings, farmers may have few feasible alternatives because of the meagre profit they receive from farming, especially if they may fail to provide for themselves and their families without using these measures for disease treatment and prevention (Pokharel *et al.*, 2024). The coercive action may not be justified in cases like this, unless it comes with additional supports (for instance, livestock insurance programmes) which create the conditions for agents to have feasible alternatives, as is needed to establish moral responsibility (Boden and Mellor, 2020). Thus, this ethical justification for coercive AMS policy may be more appropriately applied in HIC settings than LMIC settings, and it relies on the establishment of moral responsibility of the group being held accountable or able to create change in antibiotic use.

### Harm Principle

The harm principle is often appealed to as a justification for third parties intervening in individuals’ affairs. Mill’s original formulation of the harm principle (1859) has been adapted into a positive argument for intervention more recently, acting as a justification for criminal justice (Feinberg, 1984) and to justify coercive actions to mitigate climate change (Shue, 1999). The argument goes that it is justified for a third party to intervene if and only if it is to prevent an individual from performing an action that will cause (significant) harm to others. In the AMR context, this has been used to argue for interventions on those who carry resistant bacteria, such as eradication or removal of the resistant strains through invasive or non-invasive bactericidal treatment (Jamrozik and Selgelid, 2020), and for restricting farmers’ access to human-critical antibiotics for livestock (Johnson, forthcoming). The former interventions also require surveillance to detect and track the health of carriers of resistant bacteria, who may often be, themselves, asymptomatic.

Arguments from the harm principle are clearly limited in relying on evidence of prospective harm to another from one person’s actions. It may be difficult to formulate coercive actions according to such an individualistic principle, as policies will often apply on a group level that does not allow for such fine-scale differentiation and investigation of individual threats of harm (Nuffield Council on Bioethics, 2007). What’s more, there may be multiple sources of harm leading to multiple potential state actions which must be weighed against each other—for instance, the threat of COVID-19 spread and the threat of resistance, which might require conflicting measures to be taken wherein combating COVID-19 may work against efforts to prevent people from being harmed by AMR (Johnson, 2021). In contexts where individual-level data is hard to come by, rather than focussing on individual-level harms, broad coercive actions might better employ ethical justifications based on threats to population health or the public interest in protection against AMR, which might refer to the effectiveness and tragedy of the commons arguments presented above.

### Paternalism

In some cases, paternalist justifications of coercive action look very similar to justifications based on the harm principle. Whilst Mill’s initial formulation of the harm principle was intended to challenge actions on the basis of paternalism (1859), many of the public health interventions we accept today come from paternalist mandates.^4^ Similarly, some justifications for coercive measures such as the eradication of harmful microbes in a person and isolation measures discussed above might rely on paternalist arguments—indeed, paternalism might be thought stronger justification than most, given that the goal is to benefit the coerced individual. At the individual level, a paternalist position would hold that action to coerce someone is justified where the intent is to benefit them and where this benefit is likely to occur through the coercive action.

The argument relies on the premise that it is beneficial for people to not be exposed to resistant pathogens. These benefits are clear in most cases. However, there are also plenty of asymptomatic, non-pathological carriers of resistant pathogens. A paternalist justification for eradicating the harmful microbes in these individuals does not hold, as they are not necessarily harmed by hosting the bacteria (though they may pose harm to others). History also contains a warning that should limit the use of paternalist justifications for coercive AMS policy. Paternalism has been used as a guise for colonialist and imperialist activities. For instance, in some African countries under colonial rule in the early 20th century, antibiotics were imported from European countries. In Uganda under British rule, frequent antibiotic use was encouraged in veterinary medicine and farming as a matter of ‘social progression’ and ‘projects of modernization’ to be contrasted with ‘ineffective’ ‘native’ management (Kayendeke *et al*., 2023). The justification for this action was often presented as benefiting farmers and local populations—a paternalist justification. However, in colonial countries across Africa it was in fact an effective means of asserting the authority of the colonisers, decreasing farmers’ reliance on traditional methods of disease control whilst increasing reliance on European methods, and a means of expanding commercial livestock farming for export to Europe (Brown and Gilfoyle, 2010). Paternalist justifications of coercive action against AMR today must not similarly act as a guise for imposing methods and values in global agriculture. As such, this justification is limited both by evidence requirements (for prospective benefit to the targeted individual or population), and by warnings about historical abuse of the concept and the potential for these to be perpetuated through future coercive AMS policy. This might be avoided through procedures that counteract authoritarian paternalist action by states, including through community consultation and democratic deliberative methods. The use of these methods, then, might be a requirement for the appropriate application of this ethical justification.

### Justice and Development

The worst consequences of AMR primarily befall the worst off, and are likely to continue to do so (Reid, 2020). Those living in countries with less well-resourced health care systems will be less able to access treatment for resistant infections and may be more likely to be exposed to them in hospital settings (Krockow and Tarrant, 2019). Those living in countries where land use and urbanisation have advanced rapidly are at greater risk of zoonotic disease outbreaks generally, stressing healthcare systems, which may lead to overcrowding, self-medication and the emergence and spread of drug resistance (Waage *et al*., 2022). What’s more, access to antibiotics as part of an ‘infrastructure for development’ has varied globally: whilst HICs’ development has benefited from access to antimicrobials for decades or centuries, the same is not true for LMICs, who are now unfairly faced with the combination of continued limited access, increasing resistance, and calls to curb their use of antibiotics further (Reid, 2020). Coercive actions to change HICs’ policies and use of antibiotics may need to be implemented by the UN in order to achieve the redistribution of resources and power that is necessary to make up for the injustices that have been imposed on some LMICs through the historical reckless use of antimicrobials in HICs (Jasovský *et al*., 2016). The SDGs provide an accepted framework for making an ethical argument for coercive action at the international level, insofar as AMR hinders progress on goals including ensuring good health and wellbeing (SDG3) and increasing decent work and economic growth (SDG8) (goals which are themselves sometimes incompatible (Taylor *et al*., 2019)). Alternatively, under a more human rights-based model, we might consider the right to health care as including the right to access to effective drugs (including antimicrobials) (Lougarre and Viens, 2021). Coercive actions may be particularly appropriate for achieving such goals because they may be fairer in applying equally to everyone in a jurisdiction, rather than allowing different levels of advantage to affect AMR-associated behaviours.

This argument for coercive action is limited in a few ways. First, there are practical questions about the remit of international organisations to coerce their member states. It may be politically difficult for organisations such as the UN to coerce their member states on some issues like AMR. Even where relationships between institutions and states or between states themselves are strong enough to ensure the enforceability of coercive measures, sustainable development may require addressing the determinants of infectious disease rather than limiting access to antibiotics (Millar, 2011). On the other hand, there are questions surrounding interstate responsibilities and the extent to which states should meddle in other states’ affairs to begin with. Under some accounts of international justice, such as nationalism, states do not have moral obligations to individuals outside their borders, and as such, an argument for coercing them for the benefit of achieving SDGs in other nations may not be considered ethically appropriate. However, we commonly hold that states have at least some obligations towards others when it comes to preserving global goods (Dwyer, 2023), and especially when it comes to mitigating risks of global catastrophe (Johnson, 2024). As such, all states should recognise this ethical justification at least to some extent, but what it requires in terms of their implementing coercive policy within their state to benefit others will depend on, at the least, their historical use of antibiotics and level of achievement of the SDGs within the jurisdiction.

### Precautionary Approach

Coercive AMS policy might be justified by reference to the precautionary approach. Many arguments from the precautionary approach applied to the AMR context come from the literature on climate change mitigation. The precautionary principle was first developed in the pursuit of environmental sustainability, stating that ‘when an activity raises threats of harm to human health or the environment, precautionary measures should be taken even if some cause and effect relationships are not fully established scientifically’ (Global Development Research Centre, 1998). This statement might apply equally to actions that *ought to be taken* to reduce risk as to actions that ought *notbe taken* to reduce risk. It might apply to actions that exacerbate or reduce climate change, and actions that exacerbate or reduce AMR (Rogers van Katwyk *et al.* 2023). There is an important premise in the precautionary approach relating to the (in)-action leading to serious harm, yet the approach also allows for scientific uncertainty surrounding the causal mechanisms relating to bad outcomes in each area. Nijsingh *et al*. (2020a) have explored the precautionary approach as applied in the AMR context with several interventions, ranging from surveillance, to drug development, to prescription policy mandates, to restricting antibiotic use in farming. Whilst they do not consider the approach to specifically recommend any of these measures without reservation, they do think it provides a good reason to take *some action.* The concept is limited by the requirement to evidence the seriousness of expected harm, which may be difficult to find, and might accord to arbitrary ideas of seriousness (Munthe, 2020). Overall, the precautionary approach may not be a very strong justification for coercive action in any contexts, though it has the advantage of not requiring as much evidence as some other justifications, as it allows uncertainty and emphasises future (rather than current) threats of harm.

### Professional Duties

The policymaker may also need to consider ethical justifications for coercive AMS policy targeted towards particular groups. Professional duties might serve to test whether such targeted policies are justified for particular professional groups. Farmers, veterinarians, scientists, drug manufacturers and others may have professional duties related to AMS. Most obviously, physicians and health care workers have AMS-related duties. Physicians’ professional duties might be used as a defence *against* prescription mandates and other AMS measures, where the argument is that physicians have a duty of care towards their individual patients that precludes them from denying their patient the best care available to treat their ailment. However, this has been objected to on the grounds that physicians have a duty of care towards all their patients, and they must rather prescribe antimicrobials judiciously and educate their patients on the risks associated with them (StopAMR, 2019) (as may need to occur with other limited resources they must allocate). Physicians and other health care professionals, then, may have a professional duty to act as antimicrobial stewards which might be enforced through coercive AMS policy.

The main limitation of this argument is that there is not always a clear, direct link between the existence of an individual moral obligation and the acceptability of coercion. It must be established both that the moral obligation exists, and that enforcing it does not infringe an individual’s rights. (Whilst this work has been done for the duty of easy rescue, these links have not been made to my knowledge with regard to these professional obligations.) Furthermore, given that all (even necessary) uses of antimicrobials may contribute to worsening AMR, it may be impossible for a physician to completely fulfil this duty: how is a physician to ensure they ‘do no harm’ whilst still prescribing antimicrobials to those who need them? There will often be a trade-off between the health of a current patient and protecting effectiveness for future patients. Finally, there may be a concern that professional duties can conflict. When surveyed, farmers hold their primary professional obligations to be contributing to food security, and ensuring animal welfare standards are upheld—AMS is only a secondary concern (Degeling and Hall, 2023). This ethical justification, then, is limited according to the specific professional duties a group has, particularly where they may conflict or where it cannot be established how the duty links to appropriate enforcement.

## Policy Implications

In the previous section, I outlined potential justifications that might feature within an ethical evaluation framework for coercive AMS policies. As indicated, these justifications each have limitations; how strong they are and whether they are applicable to justify a particular coercive AMS policy will depend both on the implementation context, specifics of the policy, and the availability of evidence to support the empirical premises.

Due to challenges in developing a comprehensive framework from these justifications (which I discuss in the next section), a full demonstration of how they apply is outside my scope here. However, I can give an indication based on previous apparent use of some of these ethical justifications in policymaking.

Consider an example. The EU Council recently banned the use of certain human-critical antibiotics in livestock for group prophylaxis, instead allowing them only for the treatment of individual animals (Council of Europe, 2018). The reasoning behind the policy is relatively explicitly stated, apparently using both arguments from effectiveness and from a tragedy of the commons. The relevant section states:

Given the limited innovation in developing new antimicrobials, it is essential that the efficacy of existing antimicrobials be maintained for as long as possible. The use of antimicrobials in medicinal products that are used in animals may accelerate the emergence and spread of resistant micro-organisms and may compromise the effective use of the already limited number of existing antimicrobials to treat human infections. Therefore, the misuse of antimicrobials should not be allowed. (Council of Europe, 2018, paragraph 44)

Note the reference to ‘compromise the effective use’ (effectiveness) and the need to protect the good of ‘efficacy of existing antimicrobials’ (commons). Note also that in the EU case there are sufficient resources to enforce the policy.^5^ How might a policymaker formulating this policy have used the ethical justifications I have presented? First, they would need to survey the range of potential justifications (supplemented by those not yet covered in the literature, which I have been unable to include here). Next, they would need to evaluate the applicability of each according to the evidence available in the EU context and the other limitations I have discussed. For instance, recall that the argument from effectiveness requires evidence of a particular action causing a bad outcome, and evidence that a coercive action may reduce the bad outcome. The policymaker would need to have this evidence. Similarly, recall that the tragedy of the commons argument is limited by the fact that the emergence of resistance against a previously effective antimicrobial is only probabilistic for each use of antimicrobials, not certain for individual cases. Yet, in the EU, there is evidence that antimicrobial use is associated with higher numbers of AMR genes in broiler chickens (Luiken *et al*., 2019), and there is evidence that, more broadly, increased country-wide use of antimicrobials in livestock correlates with resistant diseases in livestock, and decreased use correlates with decreased resistant disease (Ma *et al*., 2021). That evidence, at least, is available to the policymaker. A final limitation of both arguments is that other costs may result from a policy that is particularly effective at curbing antimicrobial use, such as additional livestock deaths,^6^ or unjust distribution of the burdens of contributing to a common good. Given that antimicrobials can still be used to treat individual sick animals, there is little livestock loss likely to be associated with the policy. Furthermore, the policy is implemented alongside other measures in other sectors, such as mandatory reporting of certain prescriptions by doctors (Dumartin, 2016). The policymaker must weigh these costs and benefits and any other applicable justifications and their limitations,^7^ and consider whether, on the whole, the choice of coercive policy is ethically justified. If it is, then they should also include the justifications used in the presentation of the policy (as can be seen in the quote above, or even better, more explicitly), to better show transparency and accountability, and to allow for public assessment of their policy choices.

## Challenges for Developing an Ethical Evaluation Framework

According to Chris Degeling *et al*., ‘Ethical frameworks can be described as pragmatic devices designed to explicate the values, principles or issues relevant to public health decisions’ (2021: 807). A common goal of such frameworks, and one that might be shared by a framework developing out of this work, is to articulate the values and priorities that ought to be considered in policymaking. In this case, the framework is to be used *prospectively*. That is, when considering what policies to implement, one aspect of evaluation would rely on the ethical justifications offered here, alongside others. The public can also make use of this framework, either to make a *post hoc* evaluation of already-implemented policies, or through a consultative process during policy development. Such consultative processes as included alongside frameworks are important, as unless the values embedded in an ethical framework are understood by and acceptable to those affected by the policy decision, their use can undermine trust in public health policy (Degeling *et al.* 2021). This speaks to the need for procedures to be put in place that create space for public analysis of policies under development, although that work is outside my scope here in presenting merely challenges for future development of the framework.

The challenges in developing the framework regard how the framework should be adapted for different contexts, and how ethical justifications should feature in the context of other priorities and guidance policymakers receive.

First, how should the justifications listed here interact, and is the answer to that question specific to particular contexts? How should uncertainty or lack of evidence affect how the framework is used? To answer this first challenge, it is important to note that the ethical justifications I have presented are not comprehensive, and would form merely a part of a fully-fledged framework. There are potential ethical justifications for coercive policy on AMS that have simply not been discussed in the bioethics literature yet. What’s more, many of the justifications that have been discussed are likely to be more important and acceptable to policymakers and publics in Western contexts, where much of the bioethics literature on the topic has been developed, so the survey of justifications as it stands is biased towards such a context. This means there is much more work to be done to create a comprehensive list that considers ethical justifications from different philosophical traditions, and to give them weightings according to the priorities within a particular public health jurisdiction.

Whilst the justifications so far also highlight the need for more research, to produce more evidence to guide policy in the area of AMS, it is important to acknowledge a general limitation in our ability to gather evidence on some of these points. For instance, difficulty may arise where there are complex causal links between resistance in the human reservoir and the emergence of resistance in animal and environmental reservoirs. A second limitation in evidence relates to the populations and geographies that have been studied to date, and where there are more resources to conduct research. Policymakers in some jurisdictions may have less evidence to act on because of these biases in the way evidence has been collected so far. In such cases, decisions will need to be made in the face of uncertainty: decisions about whether to proceed with a particular justification and policy (with or without later re-evaluation), whether to proceed on the basis only of other evidenced justifications, or whether not to proceed with the policy at all. Such decisions are necessarily highly context-dependent.

The second challenge regards how policymakers should balance the guidance offered by a fully developed ethical evaluation framework against, for example, the guidance offered by health economics, or the political pressures they experience surrounding AMS. Solving this challenge, too, lies outside my scope here, but as with all ethical frameworks, it is important to acknowledge that policymakers have normative reasons to opt for certain policies on a number of different bases, and we cannot expect economic and political concerns to be simply overridden by ethical considerations. In contexts where economic resources are more limited or where political pressures or risks of instability are greater, ethical considerations may be outweighed by other factors, and at the same time may highlight the need for reprioritisation or external support.

## Conclusion

There is still a long way to go in the development of a comprehensive ethical framework for the evaluation of coercive AMS policies. Yet, this work is important. Coercive policies may be an attractive, useful tool for policymakers to combat AMR, whilst still requiring ethical justification. In this paper, I introduced such a framework, based on ethical justifications for coercive AMS actions from the bioethics literature, and consideration of their limitations. I indicated how they might feature in policymaking in a particular context, using the example of the EU ban on use of human-critical antibiotics in farming. Further development is needed, however, for adequate ethical guidance for policymakers and publics across policy jurisdictions. This will allow policymakers to better communicate their reasoning to the public, and will allow the public to better hold policymakers to account for implementing context-appropriate, ethically acceptable coercive AMS policies.

## Footnotes

1 Note that according to some authors, public justification may require not only the presentation and acceptance of reasoned arguments for a given action, but also adequate moral character on the part of the presenter. I will set aside these questions of policymaker virtue as they are outside the scope of this work, but for more, see Donahue (2023).

2 Note that I raised other potential points against coercion earlier, including compromising peaceful relations, and failing to express consensus. Which of these hold more weight will depend on the moral culture in the policymaker’s jurisdiction. Current discussions of coercive public health measures like lockdowns in Western bioethics thoroughly investigate the role of liberty and its protection and infringement in the infectious disease context (Giubilini and Savulescu, 2020; Giubilini, 2023; Gostin, 2023; Johnson and Matlock, 2023).

3 The authors use the argument from effectiveness to compare MRSA screening with screening for multi-drug-resistant Gram-negative bacteria, and argue that the latter is less effective if implemented in the same way as MRSA screening, but may be effective if implemented differently (Nijsingh *et al*., 2020b). The argument from effectiveness has also been used to defend coercive action to address AMR generally (Littman and Viens, 2015) and specifically regarding burdensome One Health interventions (Johnson and Matlock, 2023).

4 Consider, for instance, obesity interventions, seat-belt mandates and anti-smoking laws (Anomaly, 2012; Flanigan, 2017; Wilkinson, 2021).

5 In some settings, there are gaps between the apparent coerciveness of a policy and its actual implementation, especially where there are insufficient resources to implement penalties for non-compliance. In such cases, the measures *as implemented* may be the appropriate targets of evaluation and justification, as opposed to the policies as written.

6 There is evidence that veterinarians and others are concerned about these consequences of banning metaphylaxis (Jerab *et al*., 2022).

7 How exactly justifications should be combined and weighed against each other is a topic for further work, and beyond the scope of this paper.
